# Analysis of the Formation of Scientific Communities in the Journal *Research and Education in Nursing* (2010 - 2020) and its Disciplinary Influence: an Approach from Bibliometric Analysis, Network Analysis, and Natural Language Processing

**DOI:** 10.17533/udea.iee.v42n2e12

**Published:** 2024-07-09

**Authors:** Andrés Guzmán Henao

**Affiliations:** 1 Philosopher, Master in Sociology. Professor at Universidad de Antioquia, Colombia. Email: david.guzman@udea.edu.co Universidad de Antioquia Universidad de Antioquia Colombia david.guzman@udea.edu.co

**Keywords:** research and education in nursing, academic impact, bibliometric metrics, social network analysis, scientific collaboration, natural language processing., investigación y educación en enfermería, impacto académico, métricas bibliométricas, análisis de redes sociales, colaboración científica, procesamiento de lenguaje natural, investigação e educação em enfermagem, impacto académico, métricas bibliométricas, análise de redes sociais, colaboração científica, processamento de linguagem natural

## Abstract

**Objectives.:**

This work sought to identify the academic communities that have shown interest and participation in the Journal Research and Education in Nursing and analyze the scientific impact generated by said journal.

**Methods.:**

A bibliometric analysis was carried out, as well as social network analysis and techniques of natural language processing to conduct the research. The data was gathered and analyzed during a specific study period, covering from 2010 - 2020, for articles published in the journal, and 2010 - 2022, for articles that cited the journal within Scopus. These methods permitted performing an exhaustive evaluation of the journal’s influence and reach in diverse academic and geographic contexts.

**Results.:**

During the analysis, it was noted that the journal *Research and Education in Nursing* has had significant influence in academic and scientific communities, both nationally and internationally. Collaboration networks were detected among diverse institutions and countries, which indicates active interaction in the field of nursing research. In addition, trends and emerging patterns were identified in this field, providing a more complete view of the discipline’s evolution.

**Conclusion.:**

Based on the results obtained, it is concluded that the journal *Research and Education in Nursing* has played un fundamental role in disseminating knowledge and promoting research in nursing. The combination of Bibliometric metrics, social network analysis, and natural language processing permitted utmost comprehension of its impact in the scientific and academic community globally.

## Resumen

**Objetivos.:**

Identificar las comunidades académicas que han mostrado interés y participación en la revista *Investigación y Educación en Enfermería* y analizar el impacto científico generado por esta publicación.

**Métodos.:**

Se realizó un análisis bibliométrico, así como análisis de redes sociales y técnicas de procesamiento de lenguaje natural para llevar a cabo la investigación. Los datos se recopilaron y analizaron durante un período de estudio específico, abarcando los años 2010-2020, para los artículos publicados en la revista, y 2010-2022, para los artículos que citaron la revista dentro de Scopus. Estos métodos permitieron realizar una evaluación exhaustiva de la influencia y alcance de la revista en diversos contextos académicos y geográficos.

**Resultados.:**

Durante el análisis, se observó que la revista *Investigación y Educación en Enfermería* ha ejercido una influencia significativa en las comunidades académicas y científicas, tanto a nivel nacional como internacional. Se detectaron redes de colaboración entre diversas instituciones y países, lo que indica una interacción activa en el ámbito de la investigación en enfermería. Además, se identificaron tendencias y patrones emergentes en este campo, proporcionando una visión más completa de la evolución de la disciplina.

**Conclusión.:**

Basándose en los resultados obtenidos, se concluye que la revista *Investigación y Educación en Enfermería* ha desempeñado un papel fundamental en la difusión del conocimiento y la promoción de la investigación en enfermería. La combinación de métricas bibliométricas, análisis de redes sociales y procesamiento de lenguaje natural permitió una comprensión más completa de su impacto en la comunidad científica y académica a nivel global.

## Resumo

**Objetivos.:**

Identificar as comunidades acadêmicas que demonstraram interesse e participação na revista *Nursing Research and Education* e analisar o impacto científico gerado por esta publicação colombiana.

**Métodos.:**

Foi realizada análise bibliométrica, análise de redes sociais e técnicas de processamento de linguagem natural para a realização da pesquisa. Os dados foram coletados e analisados ​​durante um período específico de estudo, abrangendo os anos 2010-2020, para artigos publicados na revista, e 2010-2022, para artigos que citaram a revista dentro do Scopus. Esses métodos permitiram uma avaliação abrangente da influência e do alcance da revista em diversos contextos acadêmicos e geográficos.

**Resultados.:**

Durante a análise, observou-se que a revista *Nursing Research and Education* tem exercido influência significativa nas comunidades acadêmica e científica, tanto nacional quanto internacionalmente. Foram detectadas redes de colaboração entre diversas instituições e países, o que indica interação ativa no campo da pesquisa em enfermagem. Além disso, foram identificadas tendências e padrões emergentes neste campo, proporcionando uma visão mais completa da evolução da disciplina.

**Conclusão.:**

Com base nos resultados obtidos, conclui-se que a revista *Nursing Research and Education* tem desempenhado um papel fundamental na divulgação do conhecimento e na promoção da investigação em enfermagem. A combinação de métricas bibliométricas, análise de redes sociais e processamento de linguagem natural permitiu uma compreensão mais completa do seu impacto na comunidade científica e académica global.

## Introduction

The journal *Research and Education in Nursing* has played a fundamental role for over four decades in disseminating research and promoting the progress of knowledge in the field of nursing. Its recognition is reflected in its indexing in prominent regional and international databases, like SciELO, Redalyc, PubMed, and Medline, as well as in its classification as Q2 in the 2023 *Scimago Journal Report*. This study centers on analyzing the academic and scientific impact generated by the journal *Research and Education in Nursing* in the disciplinary communities, especially in the field of nursing. The main objective was to identify the communities, whether institutions or countries, that have shown interest and participation in this publication. Likewise, the work seeks to trace, through the analysis of the citation impact, the academic networks that have been formed from the articles that cite the journal. To conduct this research, Bibliometric metrics, social network analysis, and natural language processing techniques were used. These tools permit carrying out an exhaustive evaluation of the journal’s influence and reach in diverse academic and geographic contexts, thus, contributing to better understand its impact in the scientific and academic community globally.

The evaluation of the quality and influence of academic journals has been traditionally guided by conventional metrics that focus on the number of citations received and the journal’s position in quartile rankings.^1^^,^^2^ However, this simplified perspective may underestimate a journal’s complexity and scope of influence, overlooking essential aspects of its contribution to the academic dialogue and to the construction of knowledge in its respective fields of study.^3^ A trend also exists among metric studies of reexamining this evaluation paradigm, highlighting the utility of bibliometric analyses as more complete and sophisticated tools to understand the dynamics of academic publications.^4^^-^^6^

In the words of Newman and Girban^7^and Blondel *et al.,*^8^ progress in computational and algorithmic capabilities have paved the road for the development of more sophisticated techniques to identify scientific communities. Among said progress, network analyses stand out, which permit identifying groupings of authors and institutions that share interests and collaborate in research.^9^ In that sense, it is crucial to acknowledge that scientific journals act as vehicles for the creation and dissemination of knowledge, and their value goes beyond simply counting citations.

From this perspective, Gabriel Vélez highlights the importance of the term "brand" in the scientific context, derived from the systems theory proposed by Luhmann.^10^ In scientific communication, brands operate as elements that allow interlocutors to orient themselves in the circulation and understanding of information. Particularly, in the setting of science, these brands play a crucial role in the production of knowledge. Each scientific publication represents an event in which multiple brands are combined to grant uniqueness and individuality to each text and the journal. The production and reproduction of scientific articles is facilitated due to the standards generated by the community, which permit identifying any member through the presence or absence of an article. These brands provide significant information for the analysis, such as the title, affiliations, the corpus and references, elements that must be comprehensible for other colleagues as in the scientific community. Citations, although extratextual, also contain significant elements that can be analyzed in semantic terms, of co-occurrence and specialization, among other aspects, thus offering a more complete vision of the network of scientific knowledge.

The brands present in articles published by journals are key indicators that permit tracing and analyzing the evolution and nature of scientific communities. These brands function as digital footprints that reveal the interconnections among authors, institutions, and subject areas within the scope of academic literature.^11^ By being identified and analyzed in systematic and quantifiable manner, these brands can provide significant signals about the structure and dynamics of the scientific collaboration network surrounding a particular journal.

In this sense, the bibliometric analysis, network analysis emerge as valuable tools that permit a more holistic evaluation of the influence and impact of a journal in the scientific community. By mapping the connections among institutions, countries, and research topics, it is possible to identify groups that share common interests and collaborate in the generation of knowledge.^5^ This approach permits a more profound understanding of the structure and cohesion of the network of scientific collaboration, as well as the identification of central and peripheral nodes within the network. Additionally, we propose integrating thematic analyses based on Natural Language Processing (NLP) techniques to identify trends and emerging patterns in nursing research.^12^ Upon applying NLP methods to the titles, abstracts, and keywords published, we can analyze systematically the themes and subthemes dealt with in the publication, which complemented with the bibliometric and network analyses provides a fuller understanding of the structure and dynamics of the field of nursing in the journal under discussion.

From the aforementioned, the principal objective of this study consisted in analyzing the scientific impact of the journal *Research and Education in Nursing* in nursing disciplinary communities. This Will be done by identifying the institutional and national communities that have been articulated around the journal’s publication, as well as tracing the academic networks formed from the articles that cite it. 

## Methods

This study focuses on analyzing the journal *Research and Education in Nursing* (IEE, for the term in Spanish), published by the Faculty of Nursing at Universidad de Antioquia (UdeA) in Colombia, with ISSN (online): 2216-0280 and ISSN (print): 0120-5307. An exhaustive data collection was conducted from 2010 to 2020, identifying 779 articles published on the journal's OJS platform and, besides, in order to see the average scope of this period, in the citation impact section, the information from 2010 to 2022 was taken up, namely, 915 articles that cited the journal in Scopus. It is important to note that the journal has been indexed in this database since 2014. The objective was to understand the reach and impact of its articles published. In the selection of primary and secondary sources, criteria such as relevance, timeliness, and pertinence for the proposed analysis were considered. 

For bibliometric analysis, detailed counts of various metrics were performed, such as the total number of articles, citations, journals, institutions, authors, countries. These bibliometric counts provided a quantitative view of the scientific production related with the journal and permitted identifying significant trends and patterns.

Moreover, data of affiliations and countries were analyzed with co-authorship network metrics through Community Detection analysis,^13^^,^^14^ which permitted detecting densely connected groups of institutions or countries; the purpose of this study was to understand which communities have grown with and in the journal these years.

The data analysis used diverse tools and libraries in Python for counting, network analysis, and natural language processing. The Natural Language Toolkit (NLTK) library was used for natural language processing tasks, like tokenization and text analysis. In addition, libraries such as Pandas were used to manipulate and structure the data collected, Matplotlib for graphical visualization of data and metrics, and Networkx for network creation and analysis. To evidence the journal’s evolution over the years, the results were divided into three periods, namely: 2010-2013, 2014-2017, and 2018-2020, and for citations: 2010-2014, 2015-2018, 2019-2022. 

The results were structured into two distinct sections. Firstly, a detailed analysis was carried out on each of the three selected periods, where the accounts of the institutions and countries that contributed the most with publications in the journal were presented. Secondly, the most cohesive communities within the journal were identified, detected via co-authorship at the institutional and national levels, and predominant themes were explored by analyzing the most recurrent n-grams and bigrams.

Once the analysis was completed at the journal level, the articles that cited the journal were examined. Due to space limitations, counts were presented of the institutions that cited the journal in the three selected periods, highlighting those that appeared more than seven times. This approach permitted identifying the most influential institutions, as well as the most relevant countries, to understand the evolution over time regarding the acceptance and participation by the communities at institutional and geographic levels.

## Results

The results of this study provide a detailed evaluation of the international level, impact, and influence of the journal *Research and Education in Nursing*. Through meticulous analysis of data collected between 2010 and 2020, significant trends in scientific production, institutional collaboration, and predominant themes within the field of nursing are outlined. Further, the journal’s impact was examined from citation data in Scopus, which encompass from 2010 to 2022, evidencing its recognition in the international scientific community. These results not only clarify the journal’s temporal evolution, but also shed light on its contribution to the academic and scientific discourse in the nursing discipline.

### Analysis of collaboration and topics by period of publication

### Period 2010 - 2013

Atop of institutions with the most articles in the journal during said period, there is Universidad de Antioquia (Colombia) with vast presence (*n* = 329), followed by Universidade de São Paulo (Brazil) (*n* = 39), Pontificia Universidad Católica de Chile (*n* = 29), Universidad Nacional de Colombia (*n* = 24), and ending with the Brazilian institution: Universidade Federal de Santa Catarina (*n* = 21); this denotes how during this period emphasis is on national universities; nevertheless, relevance of foreign universities exists, showing the journal’s commitment to increase internationalization.. 

In the analysis of country distribution of participation in the journal (Table 1), Colombia leads with an outstanding presence of 497 appearances, followed by Brazil with 251. This characteristic highlights Colombia’s influence and active participation in the journal. Brazil, despite having lower presence, continues being an important player. Countries like Chile (*n* = 56), Mexico (*n* = 46), and Spain (*n* = 36) also show considerable representation, together with others like Iran (*n* = 14), the United States (*n* = 14), and Portugal (*n* = 4), reflecting the journal’s diversity and global reach in the international academic setting. Notwithstanding, it must be indicated how the journal has a high number of Colombian authors during this period, which is also observed in the network analysis.

**Table 1:** Distribution of articles published according to country and period

Country	2010-2013 (*n*=783)	2014-2017 (*n*=783)	2018-2020 (*n*=242)	Total (*n*=1808)
Colombia	497	272	76	845
Brazil	251	518	102	871
Iran	14	15	92	121
India	N/A	18	25	43
Mexico	44	28	24	96
Spain	36	47	21	104
Chile	56	18	20	94
Argentina	2	1	6	9
Ecuador	2	2	2	6
Indonesia	N/A	N/A	1	1
Portugal	4	6	1	11
Ireland	N/A	N/A	1	1
Switzerland	N/A	N/A	1	1
Canada	1	5	1	7

The study identified 77 collaboration communities, each comprised by institutions and organizations from different countries. Among the countries with greater representation in said communities, there are Brazil, with 14; Mexico, with 10; and Colombia, with 9. This evidences significant participation by these countries in international collaboration in the field of nursing during the period analyzed. Furthermore, diverse geographic distribution is observed encompassing Latin America, Europe, Asia, and North America, indicating wide internationalization of nursing research and practice promoted by the journal from Universidad de Antioquia. Strong participation is noted from health institutions, universities, and research organizations in these collaboration communities, which evidenced a multidisciplinary and multisector approach in the advancement of nursing knowledge and practice.

From 2010 - 2013, the journal generated 10 dense communities among countries, but only three of these articulate several countries, while the other seven are communities articulated under the same country, namely, Portugal, Venezuela, Panama, Cuba, Argentina, Australia, and Canada. The other three communities show the relationship among authors with affiliations in different countries: the first community, composed of Colombia, Spain, Iran, Mexico, and Ecuador, suggests significant collaboration in Latin America and with Spain in the field of nursing; The second community, which includes Chile and the United States, evidences interaction between Latin America and the United States in this domain; the third community is made up by Peru and Brazil, which points to a specific collaboration between these two South American countries.

**Figure 1:**
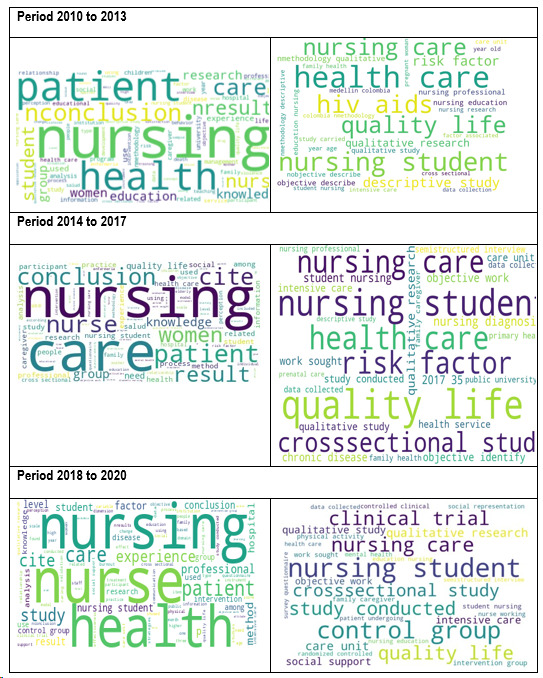
Word clouds of titles, abstracts, and keywords of articles published based on the three study periods

During the study period from 2010 - 2013 (Figure 1), the Nursing journal has addressed a vast variety of related topics, in essence, with its discipline. The topics addressed include nursing care and health practices, with research and reflections about care in different clinical and community settings, as well as strategies to improve the quality of care. In addition, nursing research has been explored, highlighting qualitative and quantitative approaches and the relationship between research methods and the professional practice. Education and teaching methods have been another focus of interest, with a detailed exam of contemporary educational approaches. Also, studies have been conducted focusing on health and medical conditions, addressing the prevalence of certain conditions, like arterial hypertension and its impact in different population groups. Lastly, the study has analyzed research and perceptions about caring for newborns, especially those cared for in neonatal units.

### Period 2014-2017

During this period, Universidad de Antioquia continues at the forefront of participation in the journal with 164 articles. However, in the remaining top five, we observe Brazilian universities (*n* = 300), revealing growth in the construction of regional communities. In the top 20, we found only four Colombian institutions: Universidad Nacional (*n* = 19), Universidad de Cartagena (*n* = 8), and the Colombian National Association of Nurses (*n* = 7); the rest are foreign institutions, mainly from Brazil, but also include institutions from Spain (*n* = 22) and the United States (*n* = 16). These findings underscore the journal’s relevance for Brazilian institutions, which consider it a reliable channel to publish and disseminate nursing topics at Latin American level.

In collaboration by countries, Brazil (Table 1) shows participation with 518 appearances, in relation to 272 by Colombia, almost duplicated; followed by Spain, Mexico, Chile, India, Iran, the United States, and Portugal, which leaves no room for doubt of the level of the journal’s internationalization role beyond the Latin American region. During the period from 2014 to 2017, the journal’s co-authorship analysis revealed a vast collaboration network that encompasses 58 communities. When comparing this period with the previous (2010-2013), an increase in observed in the number of communities identified, suggesting increased international collaboration in the field of nursing.

Among the countries most represented in these institutional communities, there are Brazil, with 17 communities; Colombia, with six communities; and Mexico, with four communities. This distribution indicates continuous and significant participation by these institutions in nursing international collaboration. Geographic diversity is also observed in the communities identified, which encompass institutions from Latin America, Europe, Asia, and North America. For the period mentioned, at country level there are eight communities, and the last five are all within the same country. The countries involved are Mexico, Iran, Ecuador, Cuba, Argentina, and Sweden.

The first community where more than one country comes together, which includes Canada, Colombia, Portugal, Philippines, Spain, Mexico, and Brazil, reflects broad and diverse collaboration in different regions of the world. The second community, composed of the United States, Colombia, Costa Rica, the United Kingdom, and Chile indicates interaction among North America, Latin America, and Europe in this domain. Lastly, the third community, comprising Australia and India, suggests collaboration between Oceania and Asia in nursing research and education.

Analysis of n-grams and bigrams (Figure 1) of the journal *Research and Education in Nursing* for the period 2014-2017 reveals prevalent topics that reflect central thematic areas in nursing. The terms "nursing" and "care" emerge as central, underlining the importance of care and nursing care. At the same time, health is positioned as a fundamental thematic axis with terms, like "health", "patients", "mental health", and "health services", indicating a comprehensive approach that encompasses general health, mental health, and health services. Furthermore, there is a marked emphasis on research and study, as evidenced in terms, like "study", "research", "data", and "objective", indicating significant dedication to the generation and analysis of knowledge in nursing. Finally, the presence of terms, like "students" and "education" suggests interest in the formation and education of future nursing professionals, highlighting the importance of academic preparation and continuous training in the field.

### Period 2018-2020

During el period analyzed, 2018-2020, Shiraz University stands out as the most active institution in scientific collaboration, with 55 appearances, followed by the National Institute of Health (*n* = 22). In third place, Universidad de Antioquia (*n* = 17). Universidad Nacional (*n =* 11) and Universidade do Rio Grande (*n* = 11) show significant presence, although with lower frequency compared with leading institutions. This change reflects a change in scientific collaboration within the journal, with Shiraz University emerging as a prominent force in research during the study period. 

In the country section, Brazil (Table 1) leads with 102 appearances, followed by Iran with 92 and Colombia with 76, which reflects strong research presence and contribution by these countries in the journal. India and Mexico also show notable presence with 25 and 24 appearances, respectively. Spain, Chile, and Argentina have significant contribution, although lower compared with the countries mentioned, with 21, 20, and 6 appearances, respectively. The presence by Ecuador, Indonesia, Portugal, Ireland, Switzerland, and Canada with one or two appearances indicates more limited, but relevant participation in the journal. Overall, said data underscore the geographic diversity of the journal’s collaborators, highlighting active contribution by countries from different regions in nursing research and practice.

During the period from 2018 to 2020, the co-authorship analysis denoted a diverse and dynamic collaboration network involving 38 communities. Compared to prior periods (2010-2013 and 2014-2017), a drop is observed in the number of communities identified, suggesting a collaboration concentration during this period. Among the countries most represented in these institutional communities, there are Iran, with 13 communities; Brazil, with six communities; and Mexico, with five communities. This distribution reflects continuous and significant participation by these countries in international collaboration in nursing, stemming from Colombia as base country. Also, diversity is noted in areas of collaboration, ranging from universities and research centers to health services and local governments. This suggests a broad range research approaches and themes within the nursing field, as well as intersectoral collaboration in the generation and application of knowledge in this setting.

In summary, the co-authorship analysis during the period 2018 - 2020 evidences continued international collaboration, although concentrated in certain countries and a diversity of fields of collaboration. This suggests evolution in nursing collaboration over time. During this period, co-authorship networks spread to nine countries listed, revealing the high scientific collaboration permitted by the journal. More countries exist in co-authorship. The first community, which includes Canada, Spain, Chile, Switzerland, Colombia, Portugal, Mexico, and Ireland reflects diverse and broad collaboration among North America, Europe, and Latin America. The second community, composed by India, Iran, and Brazil, suggests collaboration among Asia, the Middle East, and South America in this domain. Moreover, the third community, represented by Argentina, indicates an individualized approach in nursing research and education in this South American country. Finally, the fourth community, formed by Ecuador, indicates a specific interest in nursing research and education in said country.

Analysis of n-grams and bigrams (Figure 1) for the period 2018 - 2020 of the journal *Research and Education in Nursing* highlights continuous attention on "nursing" and "care", reflecting the importance of nursing care. Renewed interest is observed in formation with "students" and "education", indicating an approach on the education of future professionals. Emphasis on health is kept with "health", "patients", and "intervention", evidencing dedication to clinical intervention and treatment. Research remains essential, with terms like "study" and "research" prevailing. Additionally, there is growing attention on social and support factors, like "social" and "support", and a focus on understanding "factors" and "control" in nursing.

Briefly, the period 2018 - 2020 shows evolution in the journal central themes, maintaining its focus on nursing care, health, and research, while incorporating new focuses related with formation, social factors, and control in the field of nursing. These findings reflect emerging trends and priority areas in nursing research and practice during the period analyzed.

### Academic recognition of the journal *Research and Education in Nursing* in Scopus during the period 2010 to 2022

The journal *Research and Education in Nursing* has shown notable growth in the recognition of its articles during the period evaluated. Starting with 122 documents that cited the journal from 2010 - 2014, to the increase to 139 from 2015 - 2018 and reaching a significant number of 354 articles cited during the period 2019 - 2022. In the first period, 194 institutions appeared; 249 in the second and 652 in the third.

As displayed in Graphic 1, which only shows institutions that appear more than seven times, we note that the first period focuses attention of its articles on five institutions, three national and four international, namely, University of Miami, Pontificia Universidad Católica de Chile, Yasuj University of Medical Sciences, *Instituto de Seguridad y Servicios Sociales de los Trabajadores del Estado* (Institute of Security and Social Services of State Workers), while only one national institution stands out, Universidad de Manizales, which shows lower reach in relation to the periods that follow it, but an impact beyond its own institution. 

During the second period, eleven institutions appear over seven times, but it is noted that of the first five institutions, four are international, only one is national, namely Universidad Nacional de Colombia. The opposite of the foregoing is noted in the third period, where 49 institutions appear more than seven times, most of them international. Among the notable institutions that have cited these journals, there are Iranian universities, like Shiraz University of Medical Sciences, the Tehran University of Medical Sciences, and the Ahvaz Jundishapur University of Medical Sciences. In addition, Brazilian institutions, such as Universidade de São Paulo and Universidade Federal de São Paulo, as well as Colombian universities, like Universidad Nacional de Colombia and Universidad de Antioquia, are also in the list. At international level, presence is noted of European institutions, like the Medical University of Lublin in Poland, the University of Cambridge in the United Kingdom, and Université de Montréal in Canada. Likewise, institutions from other continents, like Vanderbilt University School of Nursing in the United States, Central South University in China, and King Saud University in Saudi Arabia, among others, have also cited the journal. This panorama suggests that the journal is gaining ground in the international academic setting, reflecting the quality and growing impact of nursing research edited in Colombia.

**Graphic 1:**
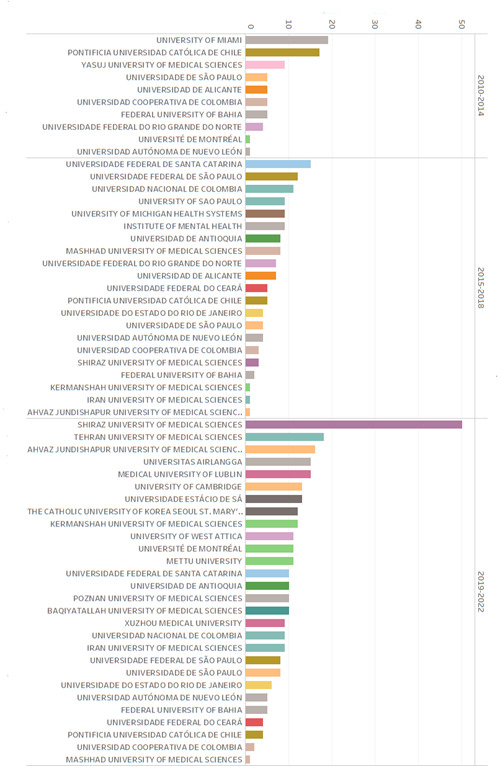
Evolution of the international recognition by institutions of the journal *Research and Education in Nursing*: 2010-2022

Upon analyzing the appearances of countries (Graphic 2) from where the journal *Research and Education in Nursing* was cited, a notable increase is identified in the internationalization of its citations over the periods studied. During the period 2010-2014, the journal was cited from 32 countries. This figure experienced an increase, reaching 36 countries during the period 2015-2018. However, the period 2019-2022 registered a significant increase, with 62 countries from where the journal is cited.

Regarding the period 2010-2014 with the countries that cited the journal in more than seven occasions, the presence of the United States, Spain, Chile, and Brazil stands out as the most prominent. Colombia occupied the fifth place in this ranking, evidencing a growing relevance of international authors during this period. During the following period, 2015-2018, Brazil emerged strongly upon registering 289 appearances, surpassing by almost six times the 49 appearances by Colombia. This was followed by Spain, Iran, the United States, and China, indicating an expansion by the journal beyond the American regional setting toward countries, like Iran and China.

Finally, in the third period analyzed, 2019-2022, Brazil maintained significant presence with 242 citations, followed by Iran with 224, China and the United States with 106 each; and finally, Italy with 88 citations. These data underline the high level of internationalization reached by the journal’s citations during this last period, consolidating its position as a publication of global relevance in the field of nursing.

### Internationalization and global relevance of the journal *Research and Education in Nursing* in Scopus 2010-2022

**Graphic 2:**
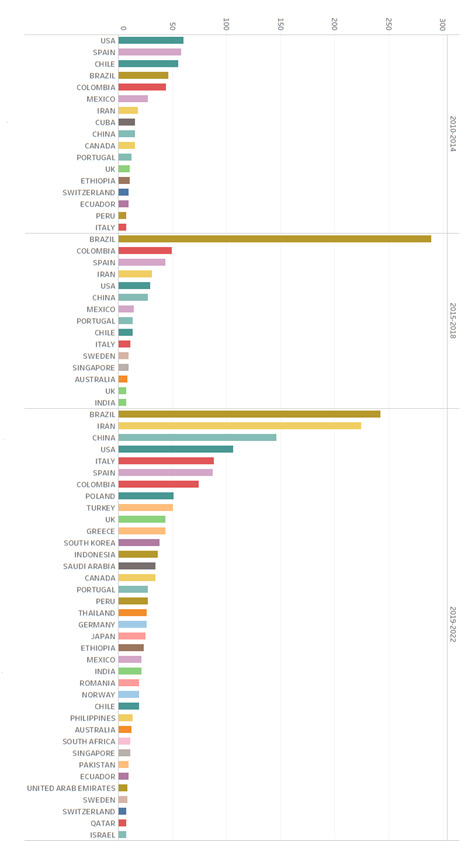
**Evolution of the international recognition by countries of the journal *Research and Education in Nursing*: 2010-2022**.

## Discussion

Starting from an approach that investigates not only metrics as final indicators, but also the ability to track diverse scientific phenomena, such as the formation of disciplinary communities through the relationships they raise, permitted observing that the journal has undergone constant change over time. Its focus has centered mainly on issues related with the reflection about the nursing practice, education in this field, research methods, and the consideration of social aspects in nursing. This trend reflects a disciplinary need in relation to research topics that encompasses not only the communities of Colombia and the region, but also countries as distant as Iran and China.

This article’s methodological approach has permitted detailing the evolution in terms of the expansion of authors who contribute to the journal, both in terms of quantity and in formation of formation of dense communities around it. Consolidation and enhancement have been observed over time, as demonstrated by the case of institutions from Brazil and from Iran. This consolidation is evidenced especially in articles that, over those 12 years, have referenced works previously published in the journal, which has contributed to the growth and in-depth discussions around certain topics.

The analysis of the evolution of the academic recognition of the journal *Research and Education in Nursing*, performed during the period analyzed, reveals a growth and consolidation trend at international level. Constant increase is observed in the good reception of the articles, with this being especially notable during the last period (2019-2022). This suggests growing recognition of the quality and relevance of the contents published in the journal.

These types of studies are not only relevant to understand the evolution and impact of an academic journal in particular, but also have significant value for the scientific community as a whole. By providing a detailed view of the internationalization, thematic diversity, and collaboration networks in a journal, these analyses not only serve to assess the quality and relevance of research in a specific field, but also offer valuable information for researchers, editors, and decision makers in the academic setting.

The importance of these types of studies lies in their capacity to identify emerging trends, highlight strong and weak areas, and guide future research and editorial policies. In addition, upon highlighting the global interconnection of the scientific community and the increasing collaboration among researchers from different countries and cultural contexts, these analyses foster the exchange of knowledge and the construction of collaborative work relations at an international level.

An important limitation of this study is its exclusive focus on the Scopus database, which could restrict full comprehension of the academic panorama in nursing. It would be beneficial in future research to conduct a comparative analysis that includes other important databases, like Web of Science (WoS) and PubMed to obtain a more complete and representative view of the journal’s position in the academic setting.

This study concludes that it may be affirmed that the exhaustive analysis conducted on the journal *Research and Education in Nursing* has yielded significant results that underscore its growth and relevance in the international academic setting. Along the years studied, the journal has experienced notable expansion in terms of its international presence, the thematic diversity of its publications, and the academic recognition of its contents.

Our study confirms that the journal has managed to consolidate itself as a leading platform for the dissemination of high-quality research in the field of nursing, attracting attention and contributions from academics from the entire world. Additionally, we have identified a clear trend toward the formation of international collaboration networks, which indicates shared commitment by the global scientific community global to advance in nursing knowledge and practice.
